# Objective Measures of Gaze Behaviors and the Visual Environment during Near-Work Tasks in Young Adult Myopes and Emmetropes

**DOI:** 10.1167/tvst.12.11.18

**Published:** 2023-11-14

**Authors:** Scott A. Read, David Alonso-Caneiro, Hosein Hoseini-Yazdi, Yan Ki Lin, Trang T. M. Pham, Rafael I. Sy, Alysha Tran, Yiming Xu, Rina Zainudin, Anjali T. Jaiprakash, Hoang Tran, Michael J. Collins

**Affiliations:** 1 Centre for Vision and Eye Research, School of Optometry & Vision Science, Queensland University of Technology, Kelvin Grove, Brisbane, Australia; 2 School of Science, Technology and Engineering, University of Sunshine Coast, Queensland, Australia; 3 Centre for Biomedical Technologies, School of Clinical Sciences, Queensland University of Technology, Kelvin Grove, Brisbane, Australia

**Keywords:** myopia, reading, eye tracking, defocus

## Abstract

**Purpose:**

To objectively quantify near-work gaze behaviors and the visual environment during reading tasks performed on a smartphone and on paper in both indoor and outdoor environments in myopes and emmetropes.

**Methods:**

A novel wearable gaze and viewing distance tracking device was used to quantify near-work gaze behaviors (focusing demand) and the visual environment (20° peripheral scene relative defocus) during a series of reading tasks. Data from nine myopes (mean age, 21 ± 1.4 years) and 10 emmetropes (21 ± 0.8 years) were analyzed. Five-minute reading tasks (matched for font type and size) were performed under four conditions: reading from a smartphone indoors, paper indoors, smartphone outdoors, and paper outdoors.

**Results:**

A significantly greater focusing demand (closer viewing distance) was found with smartphone-based reading (mean, 3.15 ± 0.74 D) compared to paper-based reading (2.67 ± 0.48 D) (*P* < 0.001), with the differences being greatest for myopic participants (*P* = 0.04). Smartphone reading was also associated with greater peripheral scene relative myopic defocus (*P* < 0.001). Although near-work behaviors were similar between environments, significantly more relative myopic defocus was found at the start of the paper-based task when performed outdoors compared to indoors (*P* = 0.02).

**Conclusions:**

Significant differences in focusing demand and scene relative defocus within a 20° field were found to be associated with reading tasks performed on a smartphone and paper in indoor and outdoor environments.

**Translational Relevance:**

These findings highlight the complex interaction between near-work behaviors and the visual environment and demonstrate that factors of potential importance to myopia development vary between paper-based and smartphone-based near tasks.

## Introduction

Myopia is the most common refractive error affecting young populations today and has emerged in recent decades as an important public health issue, with rising levels of myopia increasing the risks of a range of sight-threatening pathologies.^1^ Although genetic factors are clearly involved in the genesis of myopia,^2^ the rapid increases seen in myopia prevalence in recent years also support a strong influence of visual environmental factors.^3^ While myopia most commonly develops and progresses in childhood, it is well established that myopia can still develop and progress in young adults, particularly in university student populations and in occupations with high near-work demands.^4^ This suggests that visual environmental factors can influence myopia in children and in young adults.

Animal research has highlighted several important visual environmental factors that can impact upon eye growth and myopia development, including the type of blur/defocus experienced across the visual field as well as the intensity and spectral content of light exposure.^5^ Cross-sectional and longitudinal studies of human myopia have also identified lower levels of outdoor activities and higher levels of education as two major environmental risk factors associated with myopia.^3^ However, the specific characteristics of the visual environment that underlie these associations with human myopia are still not clear.

Although more time outdoors has consistently been associated with a lower risk of myopia development,^6^ the specific visual factors related to being outdoors underlying this association are still debated.^7^ One likely underlying mechanism may be exposure to high-intensity outdoor light and subsequent release of retinal dopamine,^8^ which is known from animal studies to slow eye growth.^9^^,^^10^ A more uniform pattern of retinal defocus across the viewing field along with less near-work activities performed in outdoor environments compared to indoor environments have also been suggested as potential visual factors linked to myopia protection.^7^^,^^11^

It has been suggested that increased near-work demands may underlie the association between education and myopia, with some studies indicating that closer working distances and/or longer periods of continuous near work are important factors associated with myopia,^12^ while other studies have not found significant associations between near work and myopia development^13^ or progression.^14^ Others have hypothesized that exposure to hyperopic defocus during near tasks may be an important factor,^15^ but evidence supporting these hypotheses to date has been inconsistent.^16^ More recently, the influence of smart devices and screen time has been proposed as a possible visual environmental factor in myopia, but evidence supporting an association between smart device use and myopia has also been mixed.^17^^,^^18^ Most studies examining the relationship between education, near activities, and myopia have relied upon questionnaires to quantify near-work activities,^3^ which may not quantify visual activities with sufficient accuracy or capture the full complexity of the modern visual environment to reliably determine these associations. Hence, a number of recent reports have recommended the development of improved methods to objectively quantify near-visual activities in order to better understand their relationship with myopia.^17^^,^^18^ An improved understanding of the visual environment and its relationship to myopia is likely to be fundamental to developing new interventions to prevent the onset and development of myopia in the future.

In recent years, studies have begun to use wearable devices that can objectively track different aspects of the visual environment that are relevant to our understanding of myopia. Studies have used wearable light sensors^19^^,^^20^ and/or distance sensors^21^^–^^25^ to quantify light exposure and/or visual task distance. The objective data from these studies have provided new insights into the visual environment and its relationship to myopia. Most studies using wearable sensors to objectively quantify visual task distance to date have used time-of-flight sensors with a relatively narrow field of view that do not take into account the wearer's gaze position within the field.^21^^–^^25^ This means the defocus profile associated with the visual environment could not be assessed by these previous studies.

Other researchers have looked to quantify the visual environment using cameras capable of mapping the distance of objects across the visual field, which allows the mapping of visual task distance as well as peripheral blur/defocus associated with the environment.^26^ Choi et al.^26^ quantified the peripheral defocus profile associated with habitual home near-work environments using a static three-dimensional (3D) distance sensor and noted a significant association between the dioptric defocus profile in the central 15° to 20° and myopia progression in childhood. However, since this analysis of defocus involved a static image of the home environment, the interaction between gaze patterns and the environment during different visual tasks was not examined. Since the defocus experienced will be dependent upon the observer's dynamic gaze position as well as the relative distance of objects in the environment, others have combined mobile gaze-tracking technology with wearable depth cameras/sensors to derive maps of peripheral defocus relative to gaze position, in different environments and/or tasks that can be tracked over time.^27^^–^^29^

Given the importance of understanding the role of the visual environment in the etiology of myopia and the ubiquitous use of handheld electronic devices, there is a need to objectively quantify the visual demands of different near-visual tasks to better understand the complex characteristics of the visual environment. We have developed a novel system combining a commercially available mobile eye tracker with a depth (distance) camera that is capable of objectively characterizing the visual environment from the perspective of the observer's gaze in both indoor and outdoor environments. In this study, we aim to use this system to provide an improved understanding of the visual demands and exposures associated with smartphone and paper-based near tasks performed in different environments in young adult myopic and emmetropic participants.

## Methods

Twenty young adults aged 18 to 25 years (mean age, 21 ± 1.4 years) and recruited from the university student population participated in this study examining near-work behaviors (e.g., focusing demand) and exposures (e.g., peripheral scene relative defocus) associated with near tasks performed in both indoor and outdoor environments. This study was approved by the QUT human research ethics committee, and all participants provided written informed consent to participate and were treated in accordance with the Declaration of Helsinki.

All participants underwent an initial screening visit to determine their refractive error and to ensure normal ocular health and binocular and accommodative function. All participants had normal corrected visual acuity in each eye of 0.00 logarithm of the minimum angle of resolution or better and had no history or evidence of ocular disease or surgery. All were in good general health and were not taking any medications known to affect vision or accommodation. No full-time contact lens wearers were included in the study, and none of the participants were undertaking optical or pharmacologic myopia control treatment. A noncycloplegic subjective refraction was used to classify the participants as myopes (spherical equivalent refraction of ≤−0.50 D in each eye) or emmetropes (spherical equivalent refraction between +0.50 and −0.25 D). All included participants had less than 1.00 D of anisometropia and less than 0.75 D of astigmatism. None of the myopic participants had habitual spectacle corrections that were significantly over- or undercorrected (>±0.25 D) compared to their subjective refraction results.

Eligible participants had ocular biometric measurements captured using an optical biometer (Lenstar LS900; Haag Streit, Köniz, Switzerland) to assess axial length. A tape measure was also used to measure each participant's height as well as their Harmon distance (the distance from their elbow to their middle knuckle of their fist).^25^

Following screening, eligible participants then returned on two separate days between 10 am and 2 pm to perform a series of near tasks while wearing a custom-developed 3D gaze and viewing distance tracker. Myopic participants wore their habitual spectacles during the near tasks. The near tasks involved reading a passage of text from a fiction novel for a 5-minute period, which was performed on a smartphone indoors, on paper indoors, on a smartphone outdoors, and on paper outdoors. Prior to beginning each task, the angle of the viewing distance camera on the eye tracker was adjusted to match the approximate task angle employed by each participant. The indoor and outdoor reading tasks were performed on different days at approximately the same time of day in a randomized order. On each reading task day, the order of the phone and paper reading task was also randomized. The ambient illuminance at the start and end of each task was also measured using a luxmeter (Extech HD450 Datalogging light meter; Extech Instruments, Waltham, MA, USA).

The same smartphone (Sony Xperia XZ; Sony, Tokyo, Japan) and paper (white A4 matte paper) were used for all participants, and both tasks involved reading high-contrast black text on a white background of the same font (Times New Roman) and font size (height of vertical letters of 3 mm), with the same fiction novel used for all participants. For each task, participants sat in a chair in front of a table and remained seated for the duration of the task. The same table and chair were used for the indoor and outdoor tasks. The same indoor room and shaded outdoor location were used for all participants. Participants could adjust the chair as needed for comfortable posture, and to allow participants to employ their typical/habitual near-work behaviors, a headrest and reading stand were not used. Surface markers were also placed at the corners of each task surface (i.e., smartphone screen or page corners) to assist the gaze analysis software method.

To ensure that the gaze position was accurately mapped to the distance camera image, a calibration procedure was performed at the start and end of each reading task. Calibration involved the participants’ fixating upon 13 circular bull’s-eye targets (presented individually on a board placed in front of the participant) located at different visual angles and at a distance similar to the tasks of interest and in the same environment as the task (i.e., outdoors or indoors).

### 3D Gaze and Viewing Distance Tracking System

To objectively quantify near-work gaze behaviors and the associated visual environment, a wearable viewing distance and gaze-tracking system was developed integrating a commercially available mobile eye tracker with a high-resolution wide-field stereo depth camera, capable of characterizing the visual environment from the perspective of the wearer's gaze, through analyses of the output images and gaze data. The two major hardware components in the wearable viewing distance and gaze-tracking system include the Pupil Labs “Core” mobile eye tracker (Pupil Labs GmbH, Berlin, Germany) and the Intel Real Sense D415 Depth camera (Intel, Santa Clara, CA, USA). The Pupil Labs eye tracker is widely used in research applications, with an open-source software framework, as well as flexible, modular hardware with a high level of documented accuracy for gaze tracking.^30^ The eye tracker utilizes two adjustable rear-facing “eye cameras” to capture images of the pupil in each eye (recording at 120 Hz) from which measures of pupil size and gaze position are determined. The eye tracker has a third forward-facing “world camera” to which the gaze coordinates are mapped to track the wearer's gaze in the visual environment.

**Figure 1.**
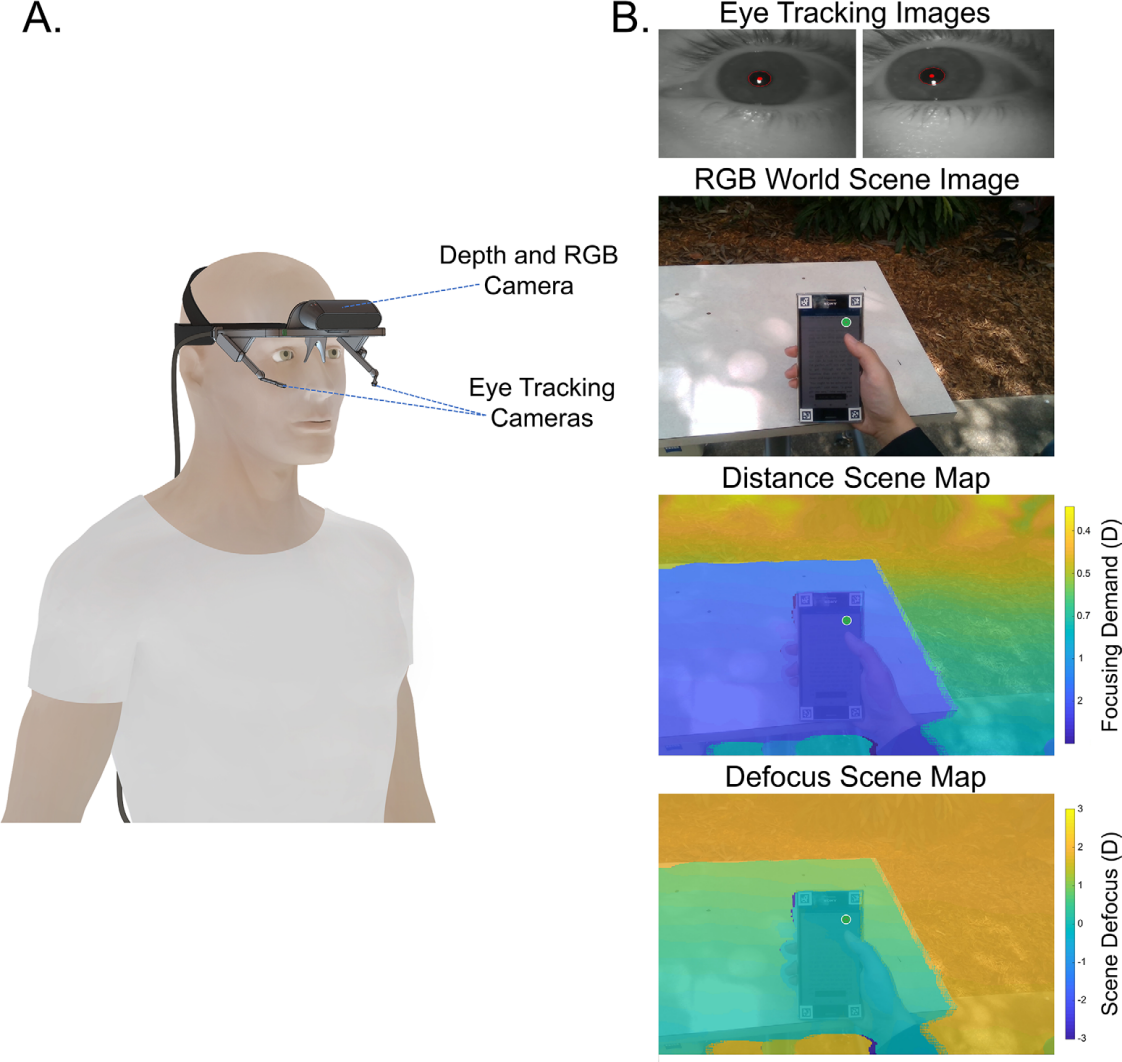
Illustration of the 3D gaze and viewing distance tracker that combines a commercially available eye-tracking system (Pupil Labs “Core” mobile eye tracker) with a depth (distance) and RGB camera (Intel Real Sense D415 Depth camera) (**A**) and example data captured during wear showing the eye-tracking camera images from which pupil size and gaze position are calculated and the RGB world scene image (note the four markers at each corner of the phone in the scene image are used to aid the gaze analysis software) and a distance scene map showing the dioptric distance of objects within the scene, as well as a scene defocus map (calculated assuming optimum focus at the point of fixation, where positive values indicate relative myopic defocus and negative values represent relative hyperopic defocus), with the gaze position (*green dot*) mapped to the visual environment (**B**). Analyses of these data allows tracking of focusing demand and scene defocus during visual tasks.

In our system, we have replaced the standard Pupil Labs world camera with a wide-field stereo depth camera, which has been attached to the Pupil Labs frame with a custom-built and angle adjustable 3D printed mount. The depth camera provides standard two-dimensional (2D) color (RGB) images of the visual environment (69° by 42°, 1920 × 1080 pixels) and associated 3D depth images (recorded at 30 Hz) calculated based upon stereo photogrammetry principles, mapping the distance of objects in the visual environment (measurement range of 0.14 to 10 m, representing a dioptric range from 0.1 to 7 D). The system connects to a laptop computer, and gaze and viewing distance data recordings are captured using the open-source Pupil Capture Software version 1.21.5 (Pupil Labs GmbH), with the plugins “Realsense2 source” and “High precision depth” activated, which support the integration of the Pupil Labs eye-tracking camera data with the data from the Intel Real Sense D415 Depth camera. Figure 1 illustrates the 3D gaze and viewing distance tracker and some example data captured by the system.

To assess the accuracy of the viewing distance measurements from the system, preliminary data were captured, with viewing distance images captured of a target that was placed at a series of known distances from the system (from 6.7 to 0.2 D) in both indoor and outdoor conditions. The viewing distance measurement from the system was compared to the known distance of the target. Analyses of these data revealed a strong correlation between the viewing distance estimation from the system and the true viewing distance (*R*^2^ > 0.99), with a mean error in object distance estimation of −0.03 ± 0.05 D (95% limits of agreement, −0.13 to 0.07 D) for indoor measures and −0.03 ± 0.11 D (95% limits of agreement, −0.24 to 0.17 D) for outdoor measures.

### Data Analysis

Following each task, the recorded gaze and viewing distance tracking videos were exported from the system and initially analyzed using the open-source Pupil Player software version 3.5.1 (Pupil Labs GmbH).^30^ Post hoc pupil detection was carried out to detect the pupil in the eye-tracking recordings of each eye, from which gaze directions were determined. Post hoc gaze calibration was then conducted based upon the calibration procedure performed at the start and end of each near task, in order to determine the function that most accurately maps the participant's gaze (pupil positions) to the reference locations in the scene (calibration markers) from the calibration procedure. The data from the calibration were then used to map the gaze location to the RGB world scene recording during the task. One participant was excluded from analyses due to poor pupil detection in their eye-tracking images (<70% of their data with valid/reliable pupil estimates), so the final analyses included 19 participants (9 myopes and 10 emmetropes). The mean accuracy of gaze mapping for all participants and all experimental conditions was 1.07° ± 0.31°. There was no significant difference in gaze mapping accuracy between myopes (mean, 1.09° ± 0.30°) and emmetropes (mean, 1.06° ± 0.33°) (*P* = 0.76). Gaze mapping accuracy also did not vary significantly between indoor (mean, 1.06° ± 0.32°) and outdoor (mean, 1.08° ± 0.40°) task environments or between the phone (mean, 1.08° ± 0.31°) and paper (mean, 1.07° ± 0.34°) reading tasks (all *P* > 0.05).

Following the preliminary analysis to map the gaze locations to the RGB world scene image, custom written software was then used to calculate metrics to characterize and track each participant's near-work gaze behaviors and visual environment during each of the 5-minute near tasks. This software initially aligned the 2D RGB scene image from the Realsense D415 camera to its associated viewing distance image, to allow the gaze coordinates for each frame to be mapped to a coordinate in the viewing distance image. The distance at the point of fixation was then converted to a dioptric demand, to allow the focusing demand of the participant to be tracked for the duration of each of the 5-minute reading tasks.

The distance at the point of fixation, in each of the recorded viewing distance images, was then used to generate a peripheral scene defocus map, to quantify the peripheral defocus across the scene relative to the participant's fixation point (i.e., the defocus in object space). This analysis assumed optimum focus at the gaze fixation position and calculated the difference between the dioptric distance at the gaze position and the dioptric distance at each of the points peripheral to the fixation point in the viewing distance image to generate a peripheral scene defocus map. Objects further away than the fixation point have positive defocus values (relative myopic defocus), whereas objects closer than the fixation point have negative defocus values (relative hyperopic defocus). The defocus map was averaged across the central 20° viewing field surrounding the gaze position (i.e., included all data points within the central 20° viewing field), to calculate the mean relative defocus associated with the scene. This was performed for each of the viewing distance images captured during the duration of the task, to track the 20° peripheral scene defocus across each of the 5-minute reading tasks. The pupil diameter of the right eye was also derived from the eye-tracking data and tracked across the duration of each task. The gaze data were also analyzed for each of the tasks to generate heatmaps of gaze locations, to understand the typical gaze behaviors employed during each of the reading tasks (with respect to the phone or paper within the scene). For each reading task, the surface markers that were placed on the corners of the phone or paper (Fig. 1) were detected in each of the scene images, to define the surface of the phone (or paper) as the region of interest for the gaze analysis, and the gaze data were then mapped onto a reference image based upon the position of the gaze relative to the region of interest.

### Statistical Analysis

The focusing demand and 20° peripheral scene relative defocus from each 60 seconds of each task were extracted from the generated data for statistical analyses. All statistical analyses were conducted using SPSS Statistics (IBM, Chicago, IL, USA). The Kolmogorov–Smirnov test revealed that the data did not depart significantly from a normal distribution (all *P* > 0.05). A repeated-measures analysis of variance (ANOVA) was used to examine whether there were significant variations in focusing demand and peripheral scene relative defocus over time, with reading task modality (paper or phone), and reading task environment (indoor or outdoor) (within-subject effects) or with refractive error group (myopes or emmetropes) (between-subject effects). To understand the potential factors influencing focusing demand and peripheral scene relative defocus, univariate regression analyses were conducted to determine the association between these parameters and demographic and biometric factors (age, axial length, pupil size, height, and Harmon distance). Bonferroni adjustments were applied to the *P* values to account for the multiple statistical comparisons.

## Results

The demographic and biometric details from the 19 participants included in the analyses are presented in the Table. As expected, the myopic participants had a significantly longer axial length than the emmetropic participants (*P* < 0.01), but the two refractive groups were well matched for age, gender balance, height, and Harmon distance (*P* > 0.05).

**Table.** Demographic and Biometric Details of the Myopic and Emmetropic Study Participants

Characteristic	Myopes (*n* = 9)	Emmetropes (*n* = 10)	*P* Values
Age, yr	21 ± 1.4	21 ± 0.8	1.0
Gender, % female	44	50	0.81
SEQ Rx, D	–2.49 ± 1.17	+0.01 ± 0.21	<0.001
AxL, mm	24.89 ± 0.85	23.79 ± 0.76	0.009
Height, cm	172 ± 14	170 ± 7	0.71
Harmon Distance, cm	37 ± 3	36 ± 2	0.51

Values are mean ± SD unless otherwise indicated. *P* values from independent samples *t*-tests comparing mean values in myopes and emmetropes are shown (apart from the effect of gender, which is the result from a chi-square test). Harmon distance is from the elbow to the middle knuckle of the fist. AxL, axial length; SEQ Rx, spherical equivalent derived from noncycloplegic subjective refraction.

**Figure 2.**
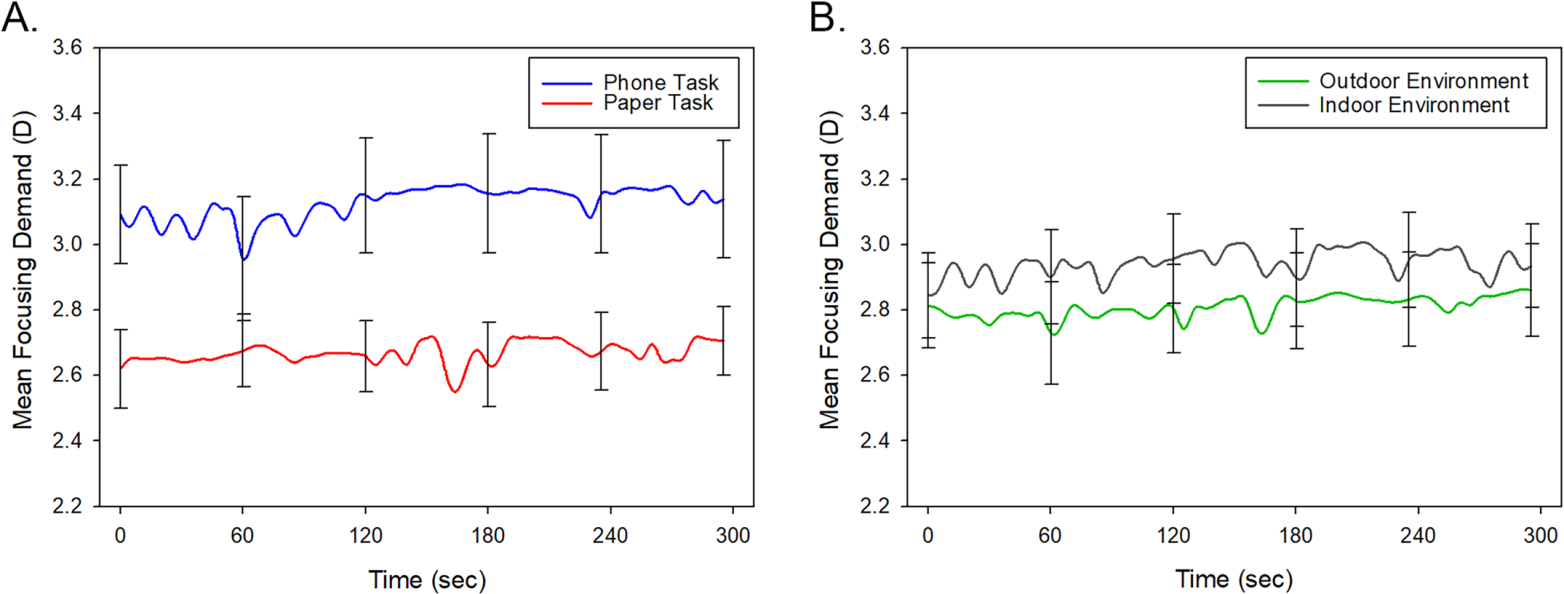
Mean focusing demand for all participants (*n* = 19) during the 5-minute reading task, when reading from a phone (averaged across indoor and outdoor environments) (*blue line*) or paper (averaged across indoor and outdoor environments) (*red line*) (**A**) and when reading in an indoor (averaged across the paper and phone tasks) (*gray line*) and outdoor (averaged across the paper and phone tasks) (*green line*) environment (**B**). *Error bars* represent the standard error of the mean.

Figure 2 illustrates the mean variations in focusing demand over time during the 5-minute reading task for the smartphone and paper tasks and for the indoor and outdoor environments. Across all tasks and environments, the mean focusing demand was 2.91 ± 0.72 D (range, 0.94 to 5.07 D). Repeated-measures ANOVA revealed a significant effect of task modality (*P* < 0.001), with a significantly greater focusing demand found when reading on a phone (mean ± standard deviation, 3.15 ± 0.74 D) compared to reading from paper (2.67 ± 0.48 D). There was no significant difference between the focusing demand between the indoor environment (2.96 ± 0.59 D) and the outdoor environment (2.86 ± 0.65 D) (*P* = 0.23) and no significant interaction between task modality and environment (*P* = 0.37). There was no significant effect of time and no significant interaction between time and task modality or environment (all *P* > 0.05), indicating that focusing demand did not vary systematically over the 5-minute reading task.

**Figure 3.**
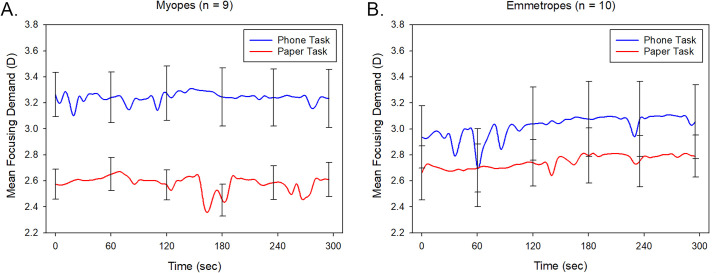
Mean focusing demand for the myopic participants (**A**) and emmetropic participants (**B**) for the 5-minute reading task, when reading from a phone (averaged across indoor and outdoor environments) (*blue line*) or paper (averaged across indoor and outdoor environments) (*red line*). *Error bars* represent the standard error of the mean.

Although there was no significant difference in the mean focusing demand during the reading tasks between the myopic (mean, 2.93 ± 0.59 D) and the emmetropic (2.89 ± 0.59 D) participants (*P* = 0.86), there was a significant refractive group-by-task modality interaction (*P* = 0.041) (Fig. 3). The myopic participants exhibited a significantly greater focusing demand for the phone task (3.28 ±0.74 D) compared to the paper task (2.59 ± 0.48 D) (*P* < 0.001), while the emmetropic participants did not exhibit a statistically significant difference between the two tasks (phone task: 3.02 ± 0.74 D, paper task: 2.75 ± 0.48 D, *P* = 0.06).

**Figure 4.**
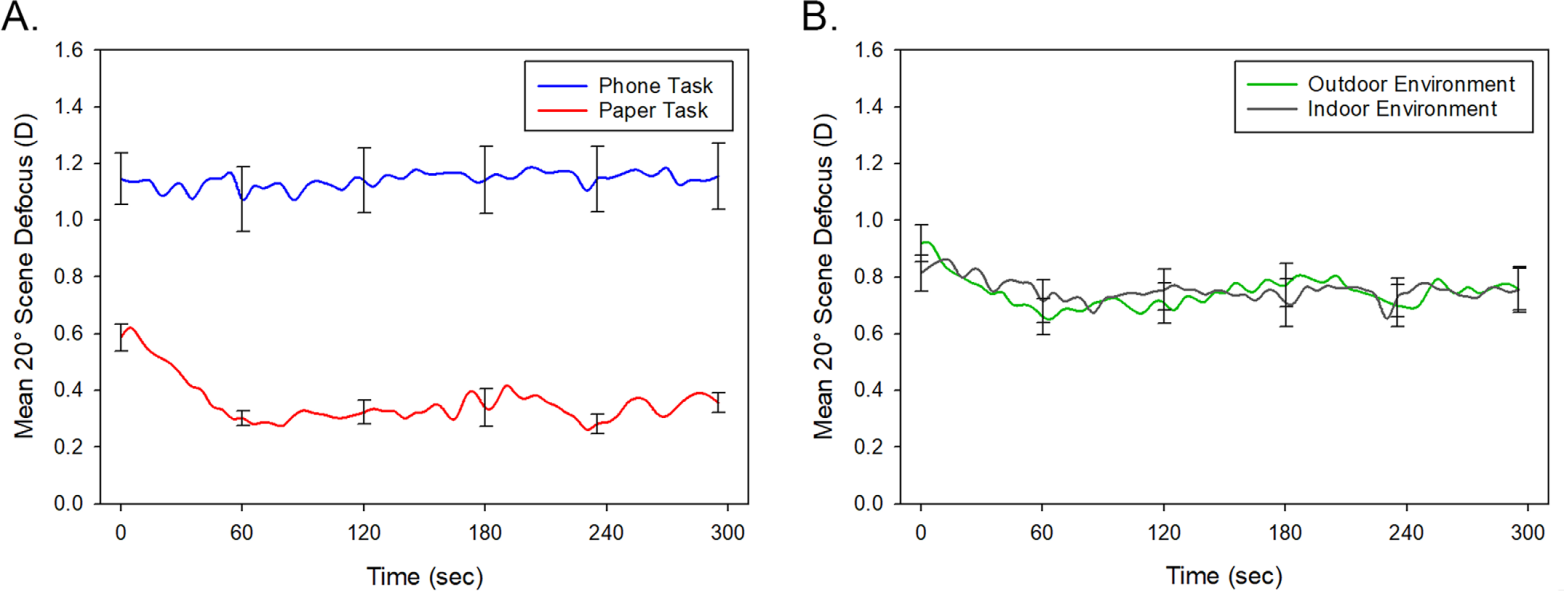
Mean 20° peripheral scene relative defocus for all participants (*n* = 19) during the 5-minute reading task, when reading from a phone (averaged across indoor and outdoor environments) (*blue line*) or paper (averaged across indoor and outdoor environments) (*red line*) (**A**) and when reading in an indoor (averaged across the paper and phone tasks) (*gray* *line*) and outdoor (averaged across the paper and phone tasks) (*green line*) environment (**B**). Positive values of scene defocus represent relative myopic defocus. *Error bars* represent the standard error of the mean.

Across all tasks and environments, the mean 20° peripheral scene relative defocus was +0.75 ± 0.57 D (range, –1.64 D to +2.37 D). Repeated-measures ANOVA for the 20° peripheral scene relative defocus data revealed significant main effects of task modality and time (both *P* < 0.001) (Fig. 4). Significantly greater peripheral scene relative myopic defocus was observed associated with the phone task (1.14 ± 0.47 D) compared to the paper task (0.37 ± 0.10 D). On average, the level of relative myopic defocus was found to be greatest at the start of the task and then reduced at subsequent time points, but these differences related to task duration were most prominent for the paper task (task modality by time interaction *P* = 0.008). Bonferroni-adjusted pairwise comparisons revealed no significant differences in 20° peripheral scene relative defocus between any of the time points for the phone task, but for the paper task, the relative defocus at the start of the reading task was significantly greater compared to all other time points (all *P* < 0.05), with the exception of the 3-minute time point (*P* = 0.06). There was no significant effect of environment for 20° scene defocus (*P* = 0.88), and no differences were observed between the refractive groups (*P* = 0.29).

**Figure 5.**
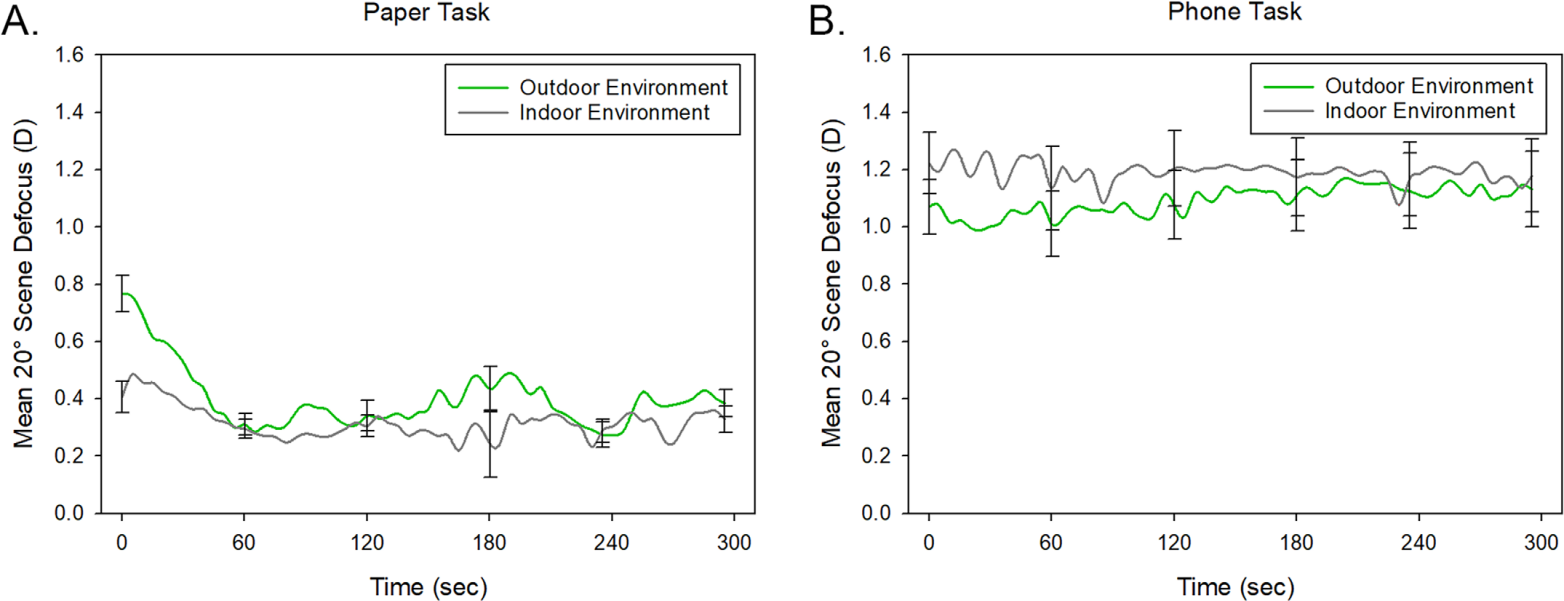
Mean 20° peripheral scene relative defocus for all participants (*n* = 19) during the 5-minute reading task when reading in outdoor (*green line*) and indoor (*gray* *line*) environments for the paper reading task (**A**) and the phone reading task (**B**). Positive values of scene defocus represent relative myopic defocus. *Error bars* represent the standard error of the mean.

A significant environment by task modality by time interaction was observed (*P* = 0.024), due to differences in 20° scene defocus for the paper reading task associated with environment at the start of the task (Fig. 5). Bonferroni-adjusted pairwise comparisons revealed significantly greater myopic defocus for the paper task performed outdoors (0.77 ± 0.28 D) compared to when performed indoors (0.40 ± 0.24 D) at the beginning of the 5-minute reading task (*P* < 0.001). For the phone task, there were no significant differences in the 20° scene defocus between the two environments at any time points (all *P* > 0.05).

As expected, the ambient illuminance was significantly greater in the outdoor environment (mean, 4133 ± 3784 lux) compared to the indoor environment (mean, 152 ± 24 lux) (*P* < 0.001). However, there was no significant difference between the ambient illuminance of the phone and paper tasks, or between the illuminance measurements captured at the start and end of the tasks (both *P* > 0.05). Consistent with the differences in illuminance between the indoor and outdoor environments, pupil size was found to be significantly greater in the indoor (mean, 3.57 ± 0.66 mm) compared to the outdoor (mean, 2.82 ± 0.56 mm) environment (*P* < 0.001). A significant difference in pupil size was also found between the phone (mean, 3.32 ± 0.70 mm) and paper tasks (mean, 3.08 ± 0.72 mm) (*P* = 0.002), but there was no significant difference associated with refractive group (*P* = 0.54).

**Figure 6.**
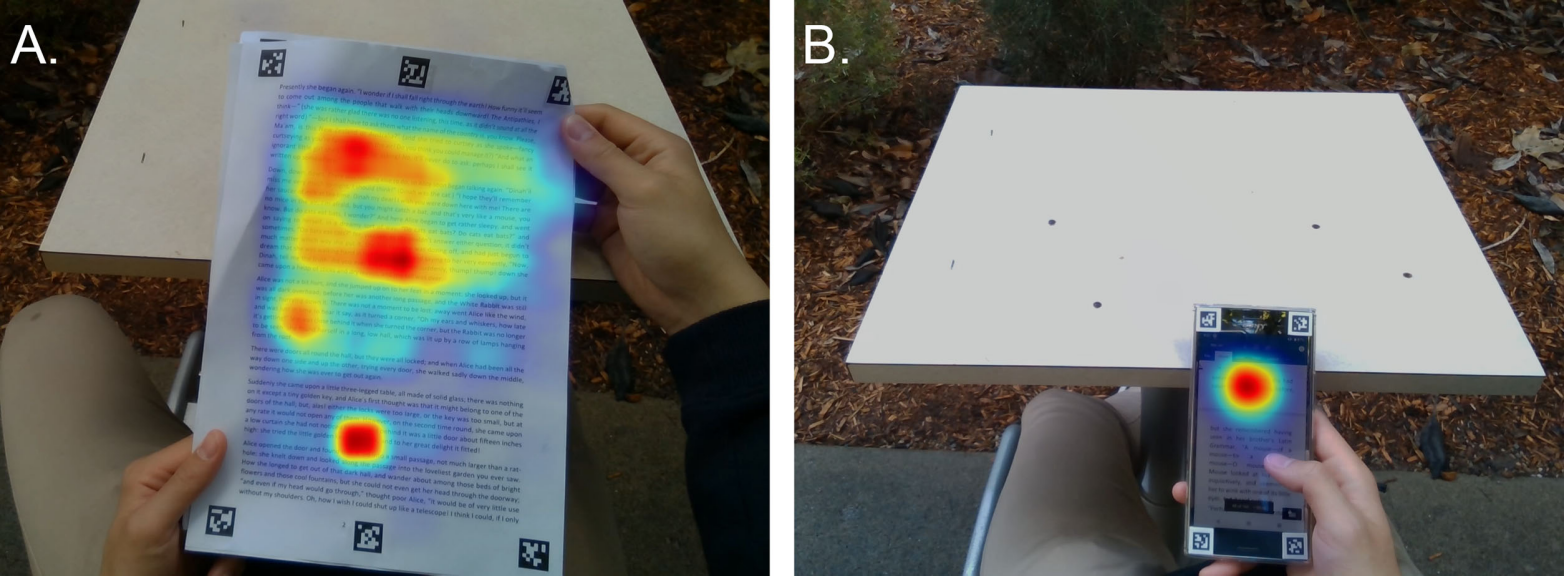
Example heatmaps of gaze density from a representative participant for the paper-based (**A**) and smartphone-based (**B**) reading tasks. Note the gaze extends across a large portion of the task surface for the paper-based reading task but is restricted to the upper portions of the surface for the phone-based task due to the ability to scroll the text with the phone.

Heatmaps of gaze throughout each of the 5-minute tasks were generated for each participant, referenced to the surface (smartphone or paper) of each task. Qualitative observation of these maps indicated some general differences in patterns of gaze between the tasks across the participants. While participants tended to extend their gaze across the majority of the vertical page during reading of the paper task, gaze was more restricted to the upper portion of the smartphone, as most participants tended to scroll through the text, which limited the vertical extent of gaze during the smartphone reading (Fig. 6).

Univariate regression analyses revealed no significant association between the mean focusing demand or 20° scene defocus with age, pupil size, axial length, Harmon distance, or height (all *P* > 0.05). To examine the potential for pupil size to explain differences in focusing demand between tasks and environments, we examined the correlation between the difference in pupil size between the paper and phone tasks with the difference in focusing demand between these tasks, and this analysis showed no significant association (*P* = 0.52). There was also no significant association between the difference in pupil size and the difference in focusing demand between the indoor and outdoor environments (*P* = 0.90). Therefore, variations in pupil size did not account for differences in focusing demand found between reading tasks and environmental conditions.

**Figure 7.**
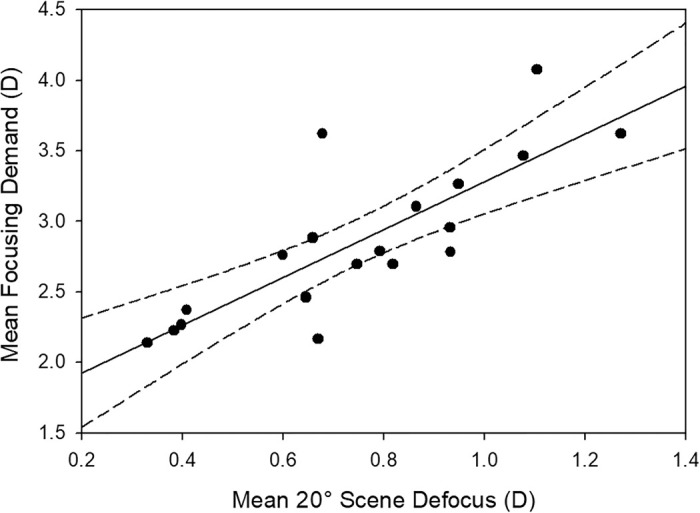
Relationship between the mean focusing demand and mean 20° scene defocus (averaged across all time points, task modalities, and environments). *Solid line* indicates best fit regression line, *dashed lines* the 95% confidence intervals.

The potential relationship between focusing demand and scene defocus was also examined. A highly significant positive association (*r* = 0.805, *P* < 0.001) was found between these parameters (Fig. 7). A greater (closer) focusing demand was associated with larger amounts of relative myopic scene defocus.

## Discussion

This study has objectively quantified near-work demands and exposures using a novel 3D-viewing distance tracker capable of mapping the dioptric distances of objects in the environment with respect to the observer's gaze. The results demonstrate significant differences in both focusing demand and peripheral scene relative defocus between smartphone-based reading and traditional paper-based reading tasks. Aspects of near-work gaze behaviors also varied between myopes and emmetropes. The visual environment also impacted upon the peripheral scene relative defocus associated with the paper-based task, with greater myopic defocus associated with reading outdoors at the start of this task. These objective data provide important new insights into the visual impacts of different visual tasks and their potential influence on myopia development.

A significant difference in focusing demand during the 5-minute reading task was observed between the smartphone and paper-based tasks, with a significantly closer working distance adopted for the smartphone reading. Consistent with our findings, previous research, documenting habitual working distance and font size with smartphones, has also noted a closer working distance in young adult participants when reading from a smartphone compared to the typical working distances employed for paper-based reading tasks, despite a similar font size being used with the two different tasks.^31^ Given that our current task involved the same reading task, matched for text size, it is likely that the difference in working distance observed in the current study is related to the physical size of the task, with participants more likely to hold the smaller smartphone closer to them than the larger paper reading task. Using a wearable distance sensor, Bhandari and Ostrin^25^ examined the working distance of children completing a series of near tasks and found that children adopted a closer working distance when playing a game on a smartphone compared to playing a game on a handheld tablet device or on paper, which also suggests that when an equivalent task is performed on two devices, the smaller device tends to be held at a closer distance.

Interestingly, the greater focusing demand during the smartphone task was most prominent in the myopic participants. Although not a universal finding,^24^ some previous studies quantifying working distance in myopic and nonmyopic participants have also found differences in working distance associated with refractive error,^21^^,^^32^ with a closer working distance in those with higher levels of myopia. Previous studies have found that closer working distances are associated with greater lags of accommodation and increased variability in accommodation responses^33^ as well as being associated with greater short-term choroidal thinning,^34^ and the cumulative effect of these factors could potentially contribute toward myopic eye growth associated with closer near tasks.

While the closer working distance employed during smartphone reading suggests a potential increased myopia risk associated with this task, the analyses of peripheral scene defocus suggest that the smartphone reading task was associated with higher levels of relative myopic defocus, which, based upon animal research,^5^ would be predicted to be protective against myopic eye growth. The potential for viewing smartphones to introduce myopic defocus into the periphery has been suggested previously,^35^ but our data have quantified these effects. Compared to the paper-based reading task, the smartphone reading task was associated with ∼0.75 D more peripheral scene relative myopic defocus. This difference in defocus appears to be due to the combined effect of the closer working distance and the smaller size of the smartphone compared to the paper task. Since the level of peripheral defocus is due to the difference in dioptric distance of the fixation point and the peripheral objects, a closer working distance will result in greater relative myopic defocus for peripheral objects located further away than the fixation point. Our analyses confirmed this significant association between closer working distances and higher levels of relative myopic scene defocus (Fig. 7). Since the entire surface of the task (i.e., the phone or the paper) will be at a similar plane of focus to the fixation point (and hence have only small levels of defocus), and the larger size of the paper task takes up a greater proportion of the central 20° analysis region, this will also contribute toward the lesser magnitude of myopic defocus with the paper-based task.

Although the smartphone task was found to exhibit greater relative myopic scene defocus, it is important to note that the retinal defocus experienced by the participant will be dependent upon the combined effects of the scene defocus, as well as the participant's natural ocular aberrations and the accuracy of their accommodation (i.e., lag of accommodation) during the task. A limitation of the current study is that these additional ocular factors were not able to be assessed during the task. Further research is needed, combining detailed optical measures of the eye and mapping of scene defocus during near tasks, to model the true levels of retinal image defocus experienced during different near tasks.

The temporal variations of the peripheral scene defocus were also different between the paper-based and smartphone reading tasks, with the defocus varying significantly over time for the paper task but remaining stable for the smartphone task. These differences appear to be a result of the gaze patterns employed during the two tasks. During the paper-based reading, relative myopic defocus was greatest at the start of the task and then reduced in magnitude. When participants begin the task, they are reading at the top of the page, which exposes a larger amount of the background of the scene to the central 20° field around fixation (analysis region), resulting in higher levels of relative myopic scene defocus. As the participants continued the reading task and their gaze moved toward the center of the page, the amount of background evident in the central 20° around fixation reduces, leading to a reduction of the level of myopic defocus. The smaller size of the phone task, and the fact that participants tended to use the touch screen to scroll through the text and direct their gaze primarily toward the upper part of the phone for the duration of the task (Fig. 6), meant that the level of relative myopic scene defocus remained relatively stable for the 5-minute smartphone task duration.

Analyses of scene defocus revealed significantly greater levels of relative myopic defocus in the outdoor environment at the start of the paper-based reading task. Greater levels of myopic defocus would be predicted to be protective against myopia. As highlighted previously, at the start of the paper-based task, participants are gazing at the top of the page, which exposes greater amounts of the central field to the background of the scene. Since in the outdoor environment, the background objects are located at a further distance compared to indoors, this resulted in a greater level of relative myopic defocus outdoors. As the task continued and gaze moved down the page, a lesser proportion of the central field was exposed to the background scene, which diminished the differences in defocus between the outdoor and indoor environments. While the greater distance of the peripheral objects in the outdoor environment might be expected to result in greater levels of myopic scene defocus outdoors for the smartphone task also, significant differences were not found. It appears that the trend for participants to employ closer working distances indoors compared to outdoors (Fig. 2B) counteracted the differences in peripheral object locations, resulting in no significant differences in peripheral scene defocus associated with environment for the smartphone task.

While these findings provide new insights into the visual demands and exposures associated with common near tasks, there are some limitations to this work. The visual tasks were performed for a relatively short duration and in controlled indoor and outdoor environments, but these conditions may not be representative of all real-world near tasks. Future work is needed to characterize longer-duration visual tasks performed in participants’ habitual environments. While our findings demonstrate differences between aspects of near-visual exposures associated with task modality and the environment, the reading task employed in this study represents only one of many different near tasks that people typically engage in for close work. Further research examining a wider variety of tasks will provide further insights.

While the gaze-tracking device was designed to fit comfortably, and participants were asked to use their typical/habitual near-work behaviors during each of the tasks, a limitation of this work (and with previous work using gaze and distance trackers to quantify near-work behaviors) is that the presence of the device and knowledge that their behavior is being assessed may have influenced their behaviors during the reading task. A further limitation is the lack of cycloplegic refraction measurements, which may influence the refractive group classification. However, it should be noted that differences between cycloplegic and noncycloplegic refraction measures are relatively small in young adults,^36^ and all myopic participants had clinically established myopia and wore spectacles habitually, which will have limited the chances of misclassification.

Our scene camera only permitted analysis of the central 20° of the visual environment. In outdoor environments, the majority of the peripheral visual field is likely to provide relative myopic defocus compared to a near-visual task, whereas for indoor environments, there is more potential for objects to be at closer distances (e.g., objects on a table/desk top) in the periphery of the visual field (i.e., more relative hyperopic defocus). However, the relative contribution of defocus in the peripheral retina versus central retina to myopia development is also a matter of conjecture.^37^

The device used in the current study provides a wealth of detailed information regarding near-work behaviors and the visual environment that was captured from young adults in a controlled research environment. Such detailed information may be valuable in understanding the impacts of visual behaviors on childhood myopia but would require measurements captured in the habitual visual environments of children during near tasks in future work. A range of improvements in the device portability (lighter weight and smaller size for more comfortable wear by children and incorporating wireless data capture to allow a more free range of activities) as well as increased robustness to device movement/slippage (that would likely be increased during use by children within their habitual environment) would be required to allow reliable data to be captured in children in their habitual environments. The minimum working distance of the current device is 14 cm, which could also impact the accuracy of measures in children employing very close near working distances.

In summary, this study demonstrates significant differences in focusing demand and scene defocus associated with reading tasks performed on a smartphone and on paper in indoor and outdoor environments. The smartphone reading task was found to have a significantly higher focusing demand (i.e., a closer working distance) but was also found to result in greater levels of peripheral scene myopic defocus compared to the traditional paper-based task. These contrasting findings highlight the complexity of the visual environment and its interaction with the visual system and is consistent with the mixed findings in the literature regarding the relationship between the use of smart devices and screen time and myopia.^17^

In conclusion, these findings highlight the complex interaction between participant behaviors, near-task modality, and the visual environment, which underscores the importance of continued work using an objective measurement method to further understand the visual exposures associated with near tasks in different environments and their potential relationship with myopia.

## References

[1] Saw S-M, Matsumura S, Hoang QV. Prevention and management of myopia and myopic pathology. *Invest Ophthalmol Vis Sci**.* 2019; 60: 488–499. 30707221 10.1167/iovs.18-25221

[2] Tedja MS, Haarman AEG, Meester-Smoor MA, et al. IMI myopia genetics report. *Invest Ophthalmol Visi Sci**.* 2019; 60: M89–M105. 10.1167/iovs.18-25965 PMC6892384 30817828

[3] Morgan IG, Wu P-C, Ostrin LA, et al. IMI risk factors for myopia. *Invest Ophthalmol Vis Sci**.* 2021; 62: 3. 10.1167/iovs.62.5.3 PMC8083079 33909035

[4] Bullimore MA, Lee SS, Schmid KL, et al. IMI-onset and progression of myopia in young adults. *Invest Ophthalmol Vis Sci*. 2023; 64: 2. 10.1167/iovs.64.6.2 PMC10153577 37126362

[5] Biswas S, Muralidharan AR, Betzler BK, et al. A duration-dependent interaction between high-intensity light and unrestricted vision in the drive for myopia control. *Invest Ophthalmol Vis Sci*. 2023; 64: 31. 10.1167/iovs.64.3.31 PMC10050902 36951855

[6] Karthikeyan SK, Ashwini DL, Priyanka M, Nayak A, Biswas S. Physical activity, time spent outdoors, and near work in relation to myopia prevalence, incidence, and progression: an overview of systematic reviews and meta-analyses. *Indian J Ophthalmol*. 2022; 70: 728–739. 35225506 10.4103/ijo.IJO_1564_21 PMC9114537

[7] Lingham G, Mackey DA, Lucas R, Yazar S. How does spending time outdoors protect against myopia? A review. *Br J Ophthalmol*. 2020; 104: 593–599. 31722876 10.1136/bjophthalmol-2019-314675

[8] Rose KA, Morgan IG, Ip J, et al. Outdoor activity reduces the prevalence of myopia in children. *Ophthalmology**.* 2008; 115: 1279–1285. 18294691 10.1016/j.ophtha.2007.12.019

[9] Stone RA, Lin T, Laties AM, Iuvone PM. Retinal dopamine and form-deprivation myopia. *Proc Natl Acad Sci USA*. 1989; 86: 704–706. 2911600 10.1073/pnas.86.2.704 PMC286542

[10] Muralidharan AR, Lança C, Biswas S, et al. Light and myopia: from epidemiological studies to neurobiological mechanisms. *Ther Adv Ophthalmol*. 2021; 13: 25158414211059246. 34988370 10.1177/25158414211059246 PMC8721425

[11] Flitcroft DI. The complex interactions of retinal, optical and environmental factors in myopia aetiology. *Prog Retin Eye Res**.* 2012; 31: 622–660. 22772022 10.1016/j.preteyeres.2012.06.004

[12] Ip JM, Saw S-M, Rose KA, et al. Role of nearwork in myopia: findings in a sample of Australian schoolchildren. *Invest Ophthalmol Vis Sci**.* 2008; 49: 2903–2910. 18579757 10.1167/iovs.07-0804

[13] Jones-Jordan LA, Sinnott LT, Manny RE, et al . Early childhood refractive error and parental history of myopia as predictors of myopia. *Invest Ophthalmol Vis Sci**.* 2010; 51: 115–121. 19737876 10.1167/iovs.08-3210 PMC2869059

[14] Saw SM, Chua WH, Gazzard G, Koh D, Tan DT, Stone RA. Eye growth changes in myopic children in Singapore. *Br J Ophthalmol*. 2005; 89: 1489–1494. 16234459 10.1136/bjo.2005.071118 PMC1772924

[15] Gwiazda J, Thorn F, Held R. Accommodation, accommodation convergence, and response AC/A ratios before and at the onset of myopia in children. *Optom Vis Sci**.* 2005; 82: 273–278. 15829855 10.1097/01.opx.0000159363.07082.7d

[16] Mutti DO, Mitchell GL, Hayes JR, et al. Accommodative lag before and after the onset of myopia. *Invest Ophthalmol Vis Sci**.* 2006; 47(3): 837–846. 16505015 10.1167/iovs.05-0888

[17] Lanca C, Saw S-M. The association between digital screen time and myopia: a systematic review. *Ophthalmic Physiol Opt**.* 2020; 40: 216–229. 31943280 10.1111/opo.12657

[18] Foremen J, Salim AT, Praveen A, et al. Association between digital smart device use and myopia: a systematic review and meta-analysis. *Lancet Digit Health**.* 2021; 3: e806–e818. 34625399 10.1016/S2589-7500(21)00135-7

[19] Read SA, Collins MJ, Vincent SJ. Light exposure and eye growth in childhood. *Invest Ophthalmol Vis Sci**.* 2015; 56: 6779–6787. 26567790 10.1167/iovs.14-15978

[20] Verkicharla PK, Ramamurthy D, Nguyen QD, et al. Development of the FitSight fitness tracker to increase time outdoors to prevent myopia. *Transl Vis Sci Technol*. 2017; 6: 20. 28660095 10.1167/tvst.6.3.20 PMC5477631

[21] Leung T-W, Flitcroft DI, Wallman J, et al. A novel instrument for logging nearwork distance. *Ophthalmic Physiol Opt**.* 2011; 31: 137–144. 21309800 10.1111/j.1475-1313.2010.00814.x

[22] Williams R, Bakshi S, Ostrin EJ, Ostrin LA. Continuous objective assessment of near work. *Sci Rep**.* 2019; 9: 6901. 31061427 10.1038/s41598-019-43408-y PMC6503122

[23] Wen L, Cao Y, Cheng Q, et al. Objectively measured near work, outdoor exposure and myopia in children. *Br J Ophthalmol*. 2020; 104: 1542–1547. 32075819 10.1136/bjophthalmol-2019-315258 PMC7587221

[24] Bhandari KR, Ostrin LA. Validation of the Clouclip and utility in measuring viewing distance in adults. *Ophthalmic Physiol Opt**.* 2020; 40: 801–814. 33002229 10.1111/opo.12735 PMC7606561

[25] Bhandari KR, Ostrin LA. Objective measures of viewing behaviour in children during near tasks. *Clin Exp Optom*. 2022; 105: 746–753. 34538208 10.1080/08164622.2021.1971049 PMC8933286

[26] Choi KY, Mok AY-T, Do C-W, Lee PH, Chan HH-L. The diversified defocus profile of the near-work environment and myopia development. *Ophthalmic Physiol Opt**.* 2020; 40: 463–471. 32519412 10.1111/opo.12698 PMC7497190

[27] Spraque WW, Cooper EA, Reissier S, Yellapragada B. The natural statistics of blur. *J Vis**.* 2016; 16: 1–27. 10.1167/16.10.23 PMC5015925 27580043

[28] Garcia MG, Ohlendorf A, Schaeffel F, Wahl S. Dioptric defocus maps across the visual field for different indoor environments. *Biomed Opt Exp**.* 2018; 9: 347–359. 10.1364/BOE.9.000347 PMC5772587 29359108

[29] Wagner P, Ho A, Kim J. Objective quantification and topographic dioptric demand of near-work. *Transl Vis Sci Technol**.* 2023; 12: 28. 10.1167/tvst.12.2.28 PMC9942781 36799872

[30] Kassner M, Patera W, Bulling A. Pupil: An open source platform for pervasive eye tracking and mobile gaze-based interaction. In: *Proceedings of the 2014 ACM International Joint Conference on Pervasive and Ubiquitous Computing: Adjunct Publication*. 2014: 1151–1160.

[31] Bababekova Y, Rosenfield M, Hue JE, Huang RR. Font size and viewing distance of handheld smart phones. *Optom Vis Sci**.* 2011; 88: 795–797. 21499163 10.1097/OPX.0b013e3182198792

[32] Hartwig A, Gowen E, Charman WN, Radhakrishnan H. Working distance and eye and head movements during near work in myopes and non-myopes. *Clin Exp Optom*. 2011; 94: 536–544. 21762395 10.1111/j.1444-0938.2011.00623.x

[33] Harb E, Thorn F, Troilo D. Characteristics of accommodative behavior during sustained reading in emmetropes and myopes. *Vis Res*. 2006; 46: 2581–2592. 16545421 10.1016/j.visres.2006.02.006 PMC1892179

[34] Woodman-Pieterse EC, Read SA, Collins MJ, Alonso-Caneiro D. Regional changes in choroidal thickness associated with accommodation. *Invest Ophthalmol Vis Sci*. 2015; 56: 6414–6422. 26444722 10.1167/iovs.15-17102

[35] Schaeffel F. Prävention der myopie [Prevention of myopia]. *Ophthalmologe*. 2019; 116: 509–517. 31069447 10.1007/s00347-019-0892-4

[36] Sanfilippo PG, Chu BS, Bigault O, et al. What is the appropriate age cut-off for cycloplegia in refraction? *Acta Ophthalmol*. 2014; 92: e458–e62. 24641244 10.1111/aos.12388

[37] Smith EL III, Campbell MC, Irving E. Does peripheral retinal input explain the promising myopia control effects of corneal reshaping therapy (CRT or ortho-K) & multifocal soft contact lenses? *Ophthalmic Physiol Opt*. 2013; 33: 379–384. 23662970 10.1111/opo.12060 PMC4696396

